# The Program “Reflexion 1”—the Condition of Intensive Formation of Metacognitive Skills in Elementary School

**DOI:** 10.3390/bs10020045

**Published:** 2020-01-30

**Authors:** Anatoly Zak

**Affiliations:** Psychological Institute of Russian Academy of Education, 125009 Moscow, Russia; jasmin67@mail.ru; Tel.: +7-(495)-451-90-48

**Keywords:** reflexive actions in solving problems, children aged 9 years, non-academic program “Reflexion 1”, extracurricular classes

## Abstract

Metacognitive skills associated with reflexive actions in solving problems are an essential condition for the successful mastering of school programs and a strong indicator of the intellectual development of primary school-aged children. The purpose of this empirical research is to study the influence of the author’s program of non-educational content, “Reflexion 1”, on the formation of reflexive actions in solving problems for children aged 9 years. Thirty-two classes were conducted (once a week, outside school hours) with the experimental group of students from September to May, according to the specified program. The results show that the lessons in the “Reflexion 1” program contribute to a significant increase in the number of children aged 9 years with substantial reflexive actions in solving problems.

## 1. Introduction 

The acceleration of scientific and technical progress requires the upgrading of professional skills for specialists. This leads to more complicated training programs at all levels of education, including primary school. Mastering more complex knowledge, skills and activities implies a higher level of metacognitive skills, as well. Inadequate development of these skills, as indicated by the results of pedagogical and psychological studies, reduces the effectiveness of education in primary school and, thus complicates the successful mastery of high school programs. Modern psychology presents a significant number of studies related to the research of metacognitive skills (see, for example, [1,2,3,4,5]). 

One of the research frameworks related to the study of metacognitive skills studies their role as conditions for increasing the effectiveness of school instruction in different classes and for different school subjects. In one direction, associated with the study of metacognitive skills, their role is as conditions to increase the effectiveness of school instruction in different classes and for different school subjects. Psychologists study the importance of metacognitive skills in teaching in primary school: the positive role of metacognitive skills for understanding scientific texts in the primary school has been researched [6], and the effectiveness of metacognitive intervention (in the form of the development of metacognitive strategy) for solving mathematical problems has been shown for third graders [7]. In other works, the importance of metacognitive skills in teaching mathematics in high school has been studied: the important role of metacognitive learning for the comprehension of eighth-graders’ graphs in the development of mathematical content has been described [8], and the positive impact of the metacognitive training of eighth-graders on mathematical thinking and mathematical reasoning has been noted [9]. This area also includes research that develops the knowledge of metacognitive skills that contribute to the self-control of students in the development of the content of conceptual texts [10], and shows the effectiveness of a metacognitive approach from teachers for the successful solution of verbal problems by sixth graders [11]. 

In another direction, the conditions for the formation of metacognitive skills are being studied. To find these conditions, researchers study the activities of the teacher: serious attention is paid to pedagogical approaches that help children plan the application of different types of thinking to various tasks and take into account the effectiveness of their thinking and change it in cases of failure [12], and effective methods are developed for teaching children ways to solve mathematical problems [13]. In other studies, the organization of training is used to create conditions conducive to the improvement of metacognitive skills: methods are developed to create a metacognitive environment while mastering elementary school subjects [14], and an active dialogue learning environment, which, along with the organization of collective discussions, plays an important role in the formation of metacognitive skills for analysis and evaluation of both the content and form of children’s discussions [15]. Another study reveals the impact of various forms of education in primary school on the development of metacognitive abilities [16]. 

It would also be useful to research the study of metacognitive skills as an indicator for assessing educational achievements [17], as well as content for monitoring learning outcomes in primary school [18,19]. Remarkable results were obtained in one study, where the relative independence of intellectual and metacognitive skills was revealed when solving problems on mathematical material [20].

The analysis of the research shows, in our opinion, that the approach to the formation of metacognitive skills through the search for solutions to problems on non-educational material by children is not sufficiently developed.

Our study is devoted to the further development of this approach.

We study metacognitive skills based on the theory of activity developed by A.N. Leontyev [21,22]. According to the provisions of this theory, metacognitive skills are actions directed by a person towards the planning, control, and evaluation of an activity. When solving problems, metacognitive skills are associated with planning, monitoring and evaluating actions to achieve the desired result.

Metacognitive skills associated with problem-solving were considered by us in line with the rational theory developed by V.V. Davydov [23,24] on two types of thinking, theoretical and empirical. According to this theory, theoretical thinking is associated with the cognition of the internal and necessary properties of objects while empirical thinking is associated with the cognition of external and random properties of objects. Based on theoretical thinking, the solution to the problem is connected not only to the external, perceived elements and their relations to its content but also to the selection of the internal relations hidden from direct perception, essential relationships. Based on empirical thinking, the task of solving a problem is connected only to external, perceived elements and their relations within the problem’s content.

Metacognitive actions within the framework of this theory include reflexive actions in solving problems. Within the framework of abstract thinking, reflexive actions are aimed at clarifying the connection between the method of actions and the specifics of building a task. In this case, the person asks oneself the question: “Why did I act so and not otherwise? Why did this method of solution apply, and not the other?” Such reflexive actions are defined in the theory of V.V. Davydov as internal and substantial since they are associated with the selection of the essential bases of the method of action.

Within the framework of empirical thinking, reflexive actions are directed only at taking into account the external features of the method of action. In this case, the person asks oneself the question: “How did I act in solving the problem? What did I specifically do, what kind of operations did I do?” Such reflexive actions are defined as external and formal since they are associated with the reflection of only the perceived features of the mode of action.

It should be noted that, earlier in our experiments [25,26,27], we studied the relationship of meaningful reflexive actions with actions to find solutions to problems. It was found that Junior and middle school students perform meaningful reflexive actions more often if the task needs to act with real objects (for example, “Tower of Hanoi” puzzle) than if the task needs to act with images of real objects (for example, spatial problems). Less often, students perform meaningful reflexive actions if the task needs to act with the designations of real objects (for example, narrative-logical problems). The spatial and narrative-logical problems are contained in the Materials and Methods section of this article. 

Our study was devoted to the study of reflexive actions in solving problems in children aged 9 years. 

The original idea of the study was to show new opportunities for initial learning in the development of reflexive actions in children. In this regard, it was necessary to find content for additional classes that developed reflexive actions more intensively than usual. For this purpose, we have developed a program of non-educational content, “Reflexion 1”.

The aim of the study, in accordance with the original idea, was to study the influence of the program “Reflexion 1” on the formation of meaningful reflexive actions (related to problem solving). For this purpose, two groups of students were compared. The first contingent (control group) during the school year (September–May) mastered only the content of the standard school program at the appointed time. The second contingent (experimental group) also during the school year (September–May) mastered the content of the standard school program at the appointed time and, in addition, in extra-curricular time (one hour once a week) from September to May, in 32 classes, mastered the content of the author’s non-curricular program “Reflexion 1”. It was assumed that, at the end of the school year, the second contingent will contain more students who are able to perform meaningful reflexive actions in solving problems of a search nature than the first contingent.

## 2. Materials and Methods

The research consisted of three stages. The first stage involved two groups of students (127 children in the control group and 131 in the experimental group) solving several exploratory problems to determine the type of reflexive actions required in solving problems. The second stage involved 32 lessons in the experimental group (one lesson per week) according to the “Reflexion 1” program for 9 year old children. The third stage involved the children from both groups solving the same exploratory problems as in the first stage. 

### 2.1. Contents of the “Reflexion 1” Program

The “Reflexion 1” program is designed to conduct 32 lessons on the basis of 16 types of non-standard problems with non-curricular content: four narrative inference problems (narrative–logical problems), four comparisons of schematic object representations (comparative problems), four spatial problems, four problems that involve movement according to specific rules (route problems). During each lesson the children solve problems of one type.

Lessons in the “Reflexion 1” program were conducted according to the following plan.

Lesson 1: comparative problems (type 1, variant 1). Lesson 2: narrative-logical problems (type 1, variant 1). Lesson 3: spatial problems (type 1, variant 1). Lesson 4: route problems (type 1, variant 1). Lesson 5: comparative problems (type 2, variants 1, 2). Lesson 6: narrative-logical problems (type 2, variants 1, 2). Lesson 7: spatial problems (type 2, variants 1, 2). Lesson 8: route problems (type 2, variants 1, 2). Lesson 9: comparative problems (type 3, variants 1,3). Lesson 10: narrative–logical problems (type 3, variants 1, 3). Lesson 11: spatial problems (type 3, variants 1, 3). Lesson 12: route problems (type 3, variants 1, 3). Lesson 13: comparative problems (type 4, variants 1, 2, 3). Lesson 14: narrative-logical (type 4, variants 1, 2, 3). Lesson 15: spatial problems (type 4, variants 1, 2, 3). Lesson 16: route problems (type 4, variants 1, 2, 3). Lesson 17: comparative problems (type 1, variants 1, 4). Lesson 18: narrative-logical (type 1, variants 1, 4). Lesson 19: spatial problems (type 1, variants 1, 4). Lesson 20: route problems (type 1, variants 1, 4). Lesson 21: comparative problems (type 2, variants 1, 5). Lesson 22: narrative-logical (type 2, variants 1, 5). Lesson 23: spatial problems (type 2, variants 1, 5). Lesson 24: route problems (type 2, variants 1, 5). Lesson 25: comparative problems (type 3, variants 1, 2, 4). Lesson 26: narrative-logical (type 3, variants 1, 2, 5). Lesson 27: spatial problems (type 3, variants 1, 3, 4). Lesson 28: route problems (type 3, variants 1, 4, 5). Lesson 29: comparative problems (type 4, variants 1, 2, 3, 4, 5). Lesson 30: narrative-logical (type 4, variants 1, 2, 3, 4, 5). Lesson 31: spatial problems (type 4, variants 1, 2, 3, 4, 5). Lesson 32: route problems (type 4, variants 1, 2, 3, 4, 5).

Each problem type had five variants of conditions: three structural versions of tasks—find an answer (1), find the question (2), find a part of the initial conditions (3) and two positional variants, to check several solutions of the problem to find the right one (4) and to check several solutions of the problem to find the wrong one (5).

In the first half of the program during each lesson children solve 1, 2 and 3 variants of one problem type; in the second half of the program, children solve 1, 4 and 5 variants of one problem type.

#### 2.1.1. Narrative-Logical Problems

The four types of narrative-logical problems (the first variant of conditions) are as follows:

Type 1, e.g.: “Don, Li and Bob swam across the river. Don swam faster than Li. Li swam faster than Bob. Who swam faster than everyone else?”

Type 2, e.g.: “The words HE, HI, DI are of different colors. Blue and pink words have the same first letter, pink and red—the same second letter. Which word is blue?”

Type 3, e.g.: “Three words were written with blue, red and gray paint: SO BE GO. The blue word is to the left of the red, and the red is to the left of the gray. What color is the word BE?”

Type 4, e.g.: “Pat, Amy and Sue sent letters: two to London, one to Boston. Pat and Amy, as well as Amy and Sue sent letters to different cities. Where did Pat send her letter?” 

Variant 2, e.g.: “Don, Li and Bob were practicing high jumps. Don jumped higher than Li. Don jumped lower than Bob”. What question can be answered considering the conditions of this problem: (a) Who jumped higher than Don? (b) What style did Bob jump in? (c) Who jumped lower than Li?

Variant 3, e.g.: “Don, Li and Bob swam across the river. Don swam faster than Li. [……… ]. Who swam faster than everyone?” What do you need to add to the conditions in order to answer the question of this problem: (a) [Bob swam faster than Don]. (b) [Bob swam as fast as Don]. (c) [Li swam faster than Bob].

Variant 4, e.g.: “Di, Lo and Jim solved the problem: “Don, Li and Bob swam across the river. Don swam faster than Li. Li swam faster than Bob. Who swam slower than everyone?” Answers: (a) Don, (b) Li, (c) Bob. Lo chose answer (a). Di, answer (b). Jim, answer (c). Who made the right choice?

Variant 5, e.g.: “Di, Lo and Jim solved the problem: “Don, Li and Bob were practicing high jumps. Don jumped higher than Lee. Don jumped lower than Bob. Who jumped higher than everyone?” Answers: (a) Don, (b) Li, (c) Bob. Lo chose answer (a). Di, answer (b). Jim, answer (c). Who made the wrong choice?

#### 2.1.2. Comparative Problems

**Figure 1:**
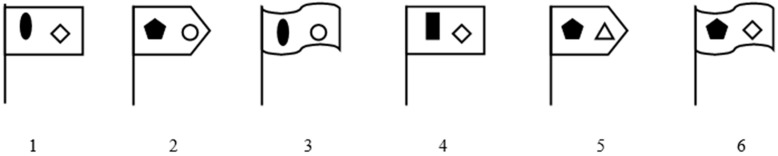
Flags.

Four types of problem for the comparison of schematic object representations (the first variant of conditions) are as follows (Figure 1): 

Type 1, e.g.: “Consider flags 2, 3, 6. Which flag is similar in shape to flag 6?”

Type 2, e.g.: “Consider flags 1, 3, 5. Which flag has an identical attribute with flag 5?”

Type 3, e.g.: “Consider flags 1, 4, 5. Which flag, 4 or 5, has more identical attributes with flag 1?”

Type 4, e.g.: “Consider flags 2, 3, 6. Which flag, 2 or 3, is similar in shape to flag 6, but has a dark figure on it similar to that of flag 1?”

During each lesson (the first and second half of the program), children (as well as in relation to logical problems) solve the same variants of each type of problem.

#### 2.1.3. Space-Combinatorial Problems.

Four types of space-combinatorial problems (the first variant of conditions) are as follows:

Type 1, e.g.: “The initial order of cards (P _ K) must be changed to their final order (K P _) for two actions.” One action is the transfer of any card to an empty seat, e.g.: action 1: (P _ K)-(_ P K), action 2: (_ P K)-(K P _).

Type 2, e.g.: “The initial order of cards (P M K) must be changed to their final order (K P M) for two actions”. One action is the mutual simultaneous movement of two cards in the initial order, for example: action 1. (P M K)-(M P K); action 2. (M P K)-(K P M).

Type 3, e.g.: “The initial order of cards—2 2 1 4 must be changed so that the same numbers are located after two actions, like the same letters—S K N N." One action is the mutual simultaneous movement of any two cards with numbers in the initial location, for example: action 1. (2 2 1 4)-(2 1 4 2); action 2. (2 1 4 2)-(4 1 2 2).

Type 4, e.g.: “How can the position of letters |M|M|S|_| be changed in two moves so that the following arrangement of digits is obtained |4|7|_|7|?”

Rule: (1) one move is the movement of any letter to a free space; (2) identical letters should be placed in the same way as identical digits.

Solution: |M|M|S|_|-|_|M|S|M|-|S|M|_|M|.

#### 2.1.4. Route Problems 

**Figure 2:**
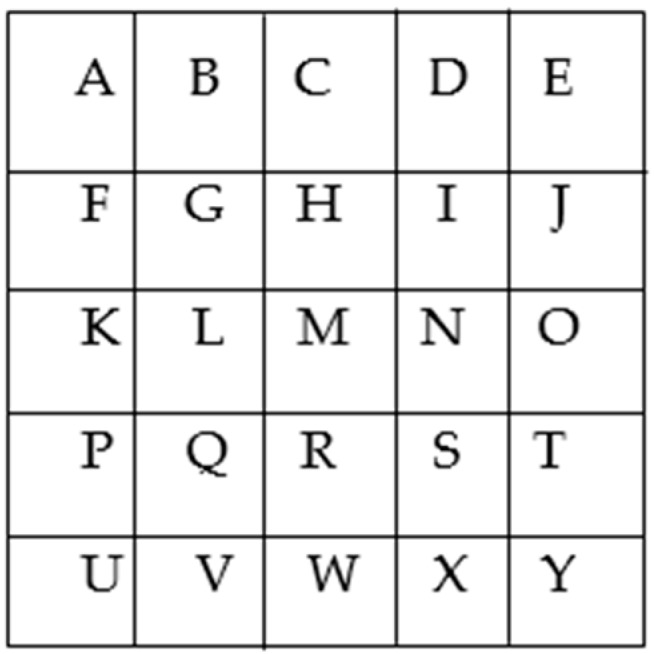
Playing field.

Four types of route problems that involve the movement of imaginary characters according to specific rules (the first variant of conditions) are as follows (Figure 2).

Type 1, e.g.: “What two steps did the duck take to get from K to R?”

Rule: (1) “Duck”, an imaginary character, moves through the letters in the cells of the square; (2) the characteristics of its movements are: (a) it steps directly, i.e., into a neighboring cell vertically (e.g.: from cell M to cell H or cell R) or horizontally (e.g.: from M to N or L); (b) it steps obliquely, i.e., diagonally, (e.g.: from M to G or I or S or Q); (3) the duck can’t make two identical steps (two direct steps or two oblique steps) in succession. Solution: K–L–R.

Type 2, e.g.: “What two jumps did the hare take to get from K to E?”

Rule: (1) “Hare”, an imaginary character, moves through the letters in the cells of the square; (2) the characteristics of its movements are: (a) it jumps directly, i.e., through the cell vertically (e.g.: from cell M to cell C or cell W) or horizontally (e.g.: from M to K or O); (b) it jumps obliquely, i.e., diagonally, e.g.: from M to E or A or U or Y; (3) hare can’t make two identical jumps (two direct jumps or two oblique jumps) in succession. Solution: K–M–E.

Type 3, e.g.: “What two moves do the duck and the hare need to make in order to get from G to T?”

Rule: (1) the duck and the hare move in turns, (2) the duck steps only directly, (3) the hare jumps only obliquely, e.g.: duck: L–G, hare: G–S, duck: S–R, hare: R–J. 

Solution: G–H–T.

Type 4, e.g.: “What two moves do the duck and the hare need to make in order to get from H to V?”

Rule: (1) the duck and the hare move in turns, (2) the duck steps only obliquely, (3) the hare jumps only directly, e.g.: duck: H–N, hare: N–D, duck: D–J, hare: J–T. 

Solution: H–L–V.

### 2.2. Enrichment Lessons

The lessons of the “Reflextion 1” program consist of three parts. During the first part (about 15 min) the teacher, together with the students, analyzes the ways of solving a typical problem. It is necessary for the children to understand what needs to be discovered in problems of this type and how this can be achieved. Children are given the means of analyzing problems and ways of managing the search for a solution and controlling their actions (for the development of reflexive actions in solving problems).

During the second part (about 30 min) the children solve 12 to 15 problems independently, applying the knowledge obtained in the first part.

During the third part (about 15 min), the teacher, along with the students, checks the solved problems and examines the incorrect solutions, once again demonstrating the methods of analyzing problems and ways of controlling mental activity.

### 2.3. Diagnostics of Reflexive Actions 

Before and after the 32 “Reflexion 1” lessons, a special lesson was held where groups of children solved alphanumeric problems wherein it was necessary to replace letters with a single number, e.g.: NG + GN = MM can be replaced by 24 + 42 = 66. First, the teacher, along with the students, analyzed the problem: T H + P = T T and explained the rules: (1) different letters are replaced by different numbers, identical letters—by identical numbers; (2) after the replacement, the correct arithmetical example should be obtained.

Then, a form was given out with two training problems:

(a) BK + M = BB    (b) DX − P = DD

The solutions of the training problems were checked during the lesson (together with the children).

Then, children were asked to solve three alphanumeric problems; the first and the third are structured identically, the second is structured in a different way.

1. AE + O = AA   2. KN + N = KK   3. ZX + W = ZZ

After the problems were solved, children were asked to select one out of five opinions related to these problems:

1. Three problems are similar because.

2. Three problems are different because.

3. Problems 1 and 2 are similar, and problem 3 is different from them because.

4. Problems 1 and 3 are similar, and problem 2 is different from them because.

5. Problems 2 and 3 are similar, and problem 1 is different from them because.

Prior to choosing an opinion, the teacher instructed the children: “On the sheets in front of you there are five different opinions about the three problems that you’ve just solved. Many children solved these problems. Some of them said that all of the problems are similar, others that all of the problems are different.”

Children in another group said that problems 1 and 2 are similar, and problem 3 is different from them. Children from yet another group said that problems 1 and 3 are similar, and problem 2 is different from them. Children from the third group said that problems 2 and 3 are similar, and problem 1 is different from them.

All children have different opinions. Each student has to choose only one opinion that he considers the most accurate, and to explain his or her choice”.

The solution of these problems and the choice of the opinion during the lesson were not evaluated.

While evaluating the results of solving the three problems after the lesson, the opinion chosen by the student and his explanation of it was taken into account.

Some of the children chose opinion 1, justifying it as follows: “... because in all of the problems letters have to be replaced by numbers”.

Some of the children chose opinion 2, justifying it as follows: “... because in all of the problems the letters are different”.

Some of the children chose opinion 3, justifying it as follows: “... because the letters in the third problem are the last in the alphabet, and it’s different in the first two problems...”.

Some of the children chose opinion 4, justifying it as follows: “... because in the second problem you have to add identical numbers, while it is different in the first and the third problems, where the numbers are not the same...”.

Some of the children chose opinion 5, justifying it as follows: “... because there were vowels in the first problem, and consonants in the other two problems...”.

If the student chose an opinion of 1, 2, 3, or 5, based on the similarities and differences in the extrinsic characteristics of the problem conditions, it was inferred that he has a superficial understanding of these problems, which indicates the presence of formal and external reflexive actions in the process of problem-solving.

If the student chose opinion 4, which is based on structural similarities and differences between problems, then it was inferred that he has a profound understanding of the problems, which indicated the presence of substantial and internal reflexive actions in the process of problem-solving.

## 3. Results

Table 1 presents data that characterize the changes in the number of students in the control and experimental groups who solved a different number of tasks designed to determine the type of reflexive actions in September and May.

Comparing September and May results for the control group, there were less children in the Subgroup 1 of the control group, respectively: 5.52% and 0.00%, the number of children in the Subgroup 2 decreased twice in May, respectively: 15.74% and 7.87%, the number of children in the Subgroup 3 increased by 1.57%, respectively 30.71% and 32.28%, the number of children in the Subgroup 4 increased by 10.81%, respectively: 48.03% and 59.84%. Thus, the most significant change (in particular, the increase) occurred among children who solved three tasks (subgroup 4).

Reported changes in the score of the subgroups of the control group reflect the nature of the influence of regular classes on the formation of problem-solving skills: there are no children who have not solved a single problem, the number of children who have solved one task decreases, and the number of children who have solved two and three tasks increases.

Turning to the results in the experimental group, there were no children left in the subgroup 1 of the experimental group (in May, compared with September), respectively: 7.63% and 0.00%, and there were no children left in the subgroup 2, respectively: 18.32% and 0.00%; the number in subgroup 3 decreased by 6.11%, respectively: 29.01% and 22.90%, and the number om subgroup 4 increased by 32.06%, respectively: 45.04% and 77.10%. Thus, the most considerable change (in particular, the increase) occurred among children who solved three tasks (subgroup 4).

Reported changes in the number of subgroups of the experimental group reflect the nature of the influence of regular classes and extracurricular classes in the non-academic program “Reflexion 1” on the formation of the ability to solve problems: there are no children who have not solved a single problem or have solved only one problem, the number of children who have solved two problems decreases and the number of children solving three problems increases.

A comparison of changes in the number of children in the four subgroups in the control and experimental groups shows the following. The typical nature of the changes is that, in both groups, there are no children left in May who have not solved a single task, and the most significant change (in particular, an increase in the number) occurs in the amount of children who have solved three tasks. The difference in the changes is that in the experimental group (as opposed to the control group) in May, there are no children who have solved only one task, and that the number of children who have solved two tasks decreases (unlike the control group, where the number of such children increases).

Analysis of the data allows us to understand how, as a result of a year’s training, the number of children who solved three tasks increased in both groups by different values: in the experimental group by 32.06% (to 77.10%), and in the control group by 10.81% (to 59.84%).

Table 2 presents the results of the choice of opinions on the problems solved by the students of the control and experimental groups in September and in May. 

In May, compared with September, there were changes in the number of students who chose each of the five opinions. Thus, the number of students who preferred Opinion 1 about solving problems (“All tasks are similar”) and Opinion 2 (“All tasks are different”) decreased in both groups: in the control group, by 9.43% (from 27.86% to 18.43%) and by 7.18% (from 22.96% to 15.78%), and in the experimental group, respectively, by 18.91% (from 28.81% to 9.90%) and by 13.54% (from 25.42% to 11.88%). It is important to note that the number of children in the experimental group decreased more than in the control group. This fact testifies, in our opinion, that the “Reflexion 1” program helps the students of the experimental group master a more substantial approach to understanding the solved problems, rather than grouping them and stating the external similarity or obvious distinction of all three tasks.

In contrast to the indicated changes, the number of students who preferred Opinion 3 of the solved problems (“The first and second tasks are similar, and the third differs from them”) and Opinion 5 (“The second and third tasks are similar, and the first differs from them”) increased in both groups: in the control one, respectively, by 0.71% (from 16.39% to 17.10%) and by 2.14% (from 13.11% to 15.25%), and in the experimental group, respectively, by 1.30% (from 13.55% to 14.85% ) and by 3.56% (from 15.25% to 18.81%). It is important to note that the number of children in the experimental group decreased more than in the control group. This fact, like the previous one, indicates that “Reflexion 1” classes contribute to mastering a more substantial approach to understanding the solved tasks by students of the experimental group, which, in this case, manifests itself in grouping tasks by pointing out the external similarity of the two tasks and the allocation of one task that is different from the other two. 

It should be noted that the way the children move from a lack of grouping solved tasks to their grouping based on obvious similarity reflects, as our studies have shown [25], a crucial phase in the development of reflexive actions in younger students aimed at solving problems.

A number of students in both groups noted Opinion 4 (“The first and third tasks are similar, and the second differs from them”), the choice of which indicates substantial reflexive actions when solving problems, because in their explanations they indicated, for example, that "... the first and third tasks are solved in the same way, and the second is different". The number of students who preferred this opinion increased in both groups: in the control group by 7.96% (from 19.67% to 27.63%), and in the experimental group significantly more, by 29.87% (from 18.64% to 48.51%)—the difference in indicators 27.63 % and 48.51% are statistically significant (at *p* < 0.01). This fact confirms the research hypothesis that classes in the “Reflexion 1” program and mastering the primary curriculum are more conducive to the formation of meaningful reflexive actions than mastering the primary curriculum only.

The results presented in both tables indicate the peculiarities of the development of reflexive actions in children aged 9 years during one year of primary school. Data describing changes in solving search problems at the end of the school year (relative to its beginning) show that training in the standard school program contributes significantly less to the formation of meaningful reflexive actions in children than their training in the standard school program in unity with the development of the content of the non-educational program “Reflexion 1”.

## 4. Discussion

The conducted research supports the initial hypothesis, which stated that solving various types of the “Reflexion 1” program problems does indeed serve as a condition for the development of reflexive actions in 9 year old children.

### 4.1. Experimental Conditions

This result is attributable to the five characteristics of the problems contained in the program: their content, character, differentiation by type, structural differences, and the variety of children’s attitudes related to the problem. Non-curricular problems of exploratory nature are offered to the children. Various types of problems are used during lessons: narrative-logical, spatial, comparative and route. Students solve variably structured problems: complete conditions and a question (find an answer), incomplete conditions and a question (find a part of the conditions), complete conditions and no question (find the question). There are two types of tasks in the program: solving problems and verify given problem solutions.

The distinctive characteristics of the enrichment lessons are important for program implementation, such as their total number, frequency and regularity, the length and the structure of each lesson. A total of 32 lessons were held over a period of nine months (September to May), with one lesson per week. Each lesson lasted 60 min, and consisted of three parts: preliminary discussion (approximately 15 min); independent problem-solving (approximately 30 min); concluding discussion (approximately 15 min).

The aim of the preliminary discussion is for the children to learn the methods of analyzing the conditions of new problem types and the ways of solving them. Such a discussion is related to the development of the ability to solve problems. The purpose of the concluding discussion is for the children to understand the methods of controlling solution methods and the criteria for evaluating the result as correct or incorrect. Such discussion is related to reflexive actions’ development.

As to the characteristics of the subjects, it has to be noted that students, classes or schools in the sample group were not deliberately selected. Lessons were held for regular students in regular classes at two regular schools. Five classes from each school participated in the lessons, with an average of 25 students per class. The control group was comprised of two classes from one school and three classes from another school, and the experimental group contained three classes from the first school and two classes from the second school.

### 4.2. Scientific Value of the Study

The study provides new knowledge regarding the conditions for the development of reflexive actions in 9 year old children. This knowledge expands and refines developmental psychology concepts on the prospects of intellectual development of children of this age.

The results of the study serve as an additional argument in support of L.S. Vygotsky’s position [28] in his polemics with J. Piaget [29]. In examining the issue of the relationship between instruction and development, L.S. Vygotsky states: “…Therefore the only good kind of instruction is that which marches ahead of development and leads it” [28] (p. 188). Our study demonstrates that teacher-assisted instruction (i.e., within the limits of proximal development) contributes to a significantly more intensive reflexive actions’ development (when compared to the control group).

### 4.3. Limitations of the Study

The results of the study should be considered with due regard to the following limitations.

The first limitation is related to the makeup of the experimental and control groups. In September, 5.51% of the students in the control group were unable to solve any of the three three-digit integer problems, 15.74% solved one such problem, 30.71% solved two problems, while in the experimental group the corresponding numbers were 7.63%, 18.32% and 29.01%. We may presume that, with a different group makeup, where the results were, for example, 10%, 20% and 35% respectively, lesson efficiency would have been lower.

The second limitation is related to the teachers’ characteristics. The average elementary school experience of the 10 teachers in both experimental and control groups equaled 15–20 years. It might be the case that the lessons were held by less experienced teachers (i.e., 3–5 years), the development of reflexive actions in the experimental group children would have been less efficient.

### 4.4. Impact of the Lessons in the Program “Reflection 1”

Observations held during the sessions allowed observation of a number of changes in children’s behavior over the course of 32 lessons.

First of all, the children began to participate more actively in the preliminary discussion, and offered more versions of problem solutions. They were no longer afraid to make a mistake as they proposed their versions, due to the fact that, in these cases, the teacher did not evaluate the children or their suggestions. He analyzed the efficiency of each suggestion together with the students.

Secondly, the attitude towards the lessons has changed, particularly in children who were not able to solve one, two or three alphanumeric problems in September. They exhibited a heightened level of anxiety during the first four to six lessons, as they were afraid that they would fail. Gradually they relaxed and began participating in the discussion of problems, suggesting various solutions.

As they solved the problems independently, these children were provided with support, particularly during the first five to eight lessons. The teacher reminded them of the rules for solving a particular type of problems, and pointed out the elements of conditions that need to be taken into account. 

For a certain time, these children were unable to independently solve problems with incomplete conditions or missing questions, or to complete tasks where the solutions needed to be verified. The teacher assisted them in understanding the inaccuracy of the wrong choices.

Thirdly, the activities of those children who successfully solved three alphanumeric problems in September were also supported. They usually managed to solve problems independently and quickly, and the teacher offered them creative tasks, such as comprising problems analogous to the ones they solved. As our research [25] demonstrates, such tasks contribute to the development of cognitive and metacognitive skills, and promote creative thinking.

Discussions with teachers helped discern the impact of the enrichment sessions on the children’s intellectual behavior. First of all, the children began to reason more consistently and examine problem conditions more precisely during math lessons (which indicates the evolution of critical thinking). Secondly, they began inventing more examples of grammar rule application during their native language lessons (which indicates the evolution of creative thinking).

Thirdly, the children demonstrated increased activity in discussing the learning material during various subject lessons and exhibited a decrease in negative emotions while studying difficult subject matter during natural science lessons.

Fourthly, after two to three months of lessons, the students who solved all of the three-digit integer problems in September often requested extra material to solve problems at home.

Additionally, the teachers noted changes in their work. In particular, during math lessons they began offering the students more problems with an atypical structure—with incomplete conditions or missing questions. Besides, during other subject lessons they started to use tasks that required verifying the accuracy of given problem solutions.

In conclusion, considering the effect of classes in the program “Reflexion 1”, it should be specifically noted that this program is developed on the basis of a course of educational classes for children 6–10 years old, “Intellektika”, on non-teaching material [30,31,32,33,34]. This course has been published for almost 20 years and is independently used by teachers in many schools in different regions of Russia for the intellectual enrichment of primary education.

### 4.5. Further Research Goals

The results of the conducted study helped formulate a number of issues for further research.

First of all, we are planning to conduct an analogous study with 8 year old children, so as to characterize the effect of the “Reflexion 1” program on the development of reflexive actions in elementary school children more fully and precisely.

Secondly, we need to identify the optimal composition of exploratory problems included in the “Reflexion 1” program for each age. In particular, a different (from the present study) quantity and contents of the types of narrative–logical, spatial, route and comparative problems are of particular research and practical interest.

Thirdly, it is desirable to verify the efficiency of the tasks that involve the independent composition of problems by children for metacognitive skills’ development. It is also important to devise tasks that require children to verify given solutions of structurally incomplete problems, where the missing part of the conditions or the missing question has to be selected from several options.

Fourthly, more efficient options have to be found in regard to: (a) the temporal ratio between the aforementioned three parts of the lesson; (b) the duration of one lesson (perhaps two 40–45 min lessons each may be combined into one lesson with a 15–20 min break); (c) frequency of lessons (six or eight lessons per month, as opposed to four lessons per month, as in the present study); (d) group size (15–20 children or less per group, as opposed to 25–26 children in the present study); and (e) by the preliminary testing success rate (15%–20% or 2%–4% of children unable to solve any of the three-digit integer problems, as opposed to 7%–8% in the present study).

Furthermore, it would be interesting to introduce certain changes into the teachers’ work: (a) engage less experienced teachers; (b) instruct the teachers not only through the texts, but mainly in the course of a seminar, where they would have a chance to communicate with the author of the program, ask him questions and hear his commentaries.

Additionally, an integrated program of teaching thinking to elementary school students should be created, wherein the “Reflexion 1” program would serve as a propedeutic of a critical and creative thinking development program.

Authors should discuss the results and how they can be interpreted in perspective of previous studies and of the working hypotheses. The findings and their implications should be discussed in the broadest context possible. Future research directions may also be highlighted.

## 5. Conclusions

The study demonstrated the development of reflexive actions in 9 year old children in different conditions. When they mastered the content of the standard initial training program, the development of reflexive actions related to problem solving occurs significantly less intensively than when they master the content of the standard initial training program and the non-teaching program “Reflexion 1”.

## Figures and Tables

**Table 1:** The number of students in the control (C) and experimental (E) groups (in proportion to the total number of students in the control and experimental groups) who were unable to solve any of the three alphanumeric problems (subgroup 1), those who solved one problem (subgroup 2), those who solved two problems (subgroup 3) and those who solved three problems (subgroup 4) in September and May.

Subgroups	September		May	
	C Group, n (%)	E Group, n (%)	C Group, n (%)	E Group, n (%)
Subgroup 1	7 (5.52)	10 (7.63)	(0.00)	(0.00)
Subgroup 2	20 (15.74)	24 (18.32)	10 (7.88)	(0.00)
Subgroup 3	39 (30.71)	38 (29.01)	41(32.28)	30 (22.90)
Subgroup 4	61 (48.03)	59 (45.04)	76 (59.84)	101 (77.10)

**Table 2:** The number of students in control (C) and experimental (E) groups who solved three problems and chose the first, second, third, fourth or fifth opinion in the control (C) and experimental (E) groups in September and May.

Opinions	September		May	
	C Group, n (%)	E Group, n (%)	C Group, n (%)	E Group, n (%)
The first	17 (27.86)	17 (28.81)	14 (18.42)	10 (9.90)
The second	14 (22.96)	14 (23.74)	11 (14.47)	8 (7.93)
The third	10 (16.39)	8 (13.56)	13 (17.10)	15 (14.85)
The fourth	12 (19.67)	11 (18.64)	21 (27.64) **	49 (48.51) **
The fifth	8 (13.11)	9 (15.25)	17 (22.37)	19 (18.81)

** = *p* < 0.01.
